# Muscarinic M4 Receptors on Cholinergic and Dopamine D1 Receptor-Expressing Neurons Have Opposing Functionality for Positive Reinforcement and Influence Impulsivity

**DOI:** 10.3389/fnmol.2018.00139

**Published:** 2018-04-24

**Authors:** Anna M. Klawonn, Daniel B. Wilhelms, Sarah H. Lindström, Anand Kumar Singh, Maarit Jaarola, Jürgen Wess, Michael Fritz, David Engblom

**Affiliations:** ^1^Department of Clinical and Experimental Medicine, Linköping University, Linköping, Sweden; ^2^Department of Psychiatry and Behavioural Sciences, Stanford University, Stanford, CA, United States; ^3^Department of Medical and Health Science, Linköping University, Linköping, Sweden; ^4^Department of Emergency Medicine, Linköping University Hospital, Linköping, Sweden; ^5^Department of Neuroscience, Baylor College of Medicine, Houston, TX, United States; ^6^Molecular Signaling Section, National Institute of Health, Bethesda, MD, United States

**Keywords:** muscarinic M4 receptor, acetylcholine, dopamine D1 receptor, reward learning, impulsivity, addiction, cocaine

## Abstract

The neurotransmitter acetylcholine has been implicated in reward learning and drug addiction. However, the roles of the various cholinergic receptor subtypes on different neuron populations remain elusive. Here we study the function of muscarinic M4 receptors (M4Rs) in dopamine D1 receptor (D1R) expressing neurons and cholinergic neurons (expressing choline acetyltransferase; ChAT), during various reward-enforced behaviors and in a “waiting”-impulsivity test. We applied cell-type-specific gene deletions targeting M4Rs in D1RCre or ChATCre mice. Mice lacking M4Rs in D1R-neurons displayed greater cocaine seeking and drug-primed reinstatement than their littermate controls in a Pavlovian conditioned place preference (CPP) paradigm. Furthermore, the M4R-D1RCre mice initiated significantly more premature responses (PRs) in the 5-choice-serial-reaction-time-task (5CSRTT) than their littermate controls, indicating impaired waiting impulse control. In contrast, mice lacking M4Rs in cholinergic neurons did not acquire cocaine Pavlovian conditioning. The M4R-ChATCre mice were also unable to learn positive reinforcement to either natural reward or cocaine in an operant runway paradigm. Immediate early gene (IEG) expression (*cFos* and *FosB*) induced by repeated cocaine injections was significantly increased in the forebrain of M4R-D1RCre mice, whereas it remained normal in the M4R-ChATCre mice. Our study illustrates that muscarinic M4Rs on specific neural populations, either cholinergic or D1R-expressing, are pivotal for learning processes related to both natural reward and drugs of abuse, with opposing functionality. Furthermore, we found that neurons expressing both M4Rs and D1Rs are important for signaling impulse control.

## Introduction

Cholinergic neurons form a wide network throughout the central nervous system, including cell populations that can modulate either local or distal neuro-circuitry. The mesolimbic system is innervated by cholinergic projections arising from two brainstem nuclei: the laterodorsal tegmental nucleus and the pedunculopontine nucleus (Oakman et al., 1995; Dautan et al., 2014). Furthermore, the striatum has its own population of cholinergic interneurons, which constitutes 1%–2% of its neurons.

The cholinergic interneurons of the Nucleus Accumbens (NAc) have been studied using modern techniques. For instance, optogenetic activation of cholinergic interneurons in the NAc was shown to enhance local phasic dopamine release (Cachope et al., 2012) and exert control over medium spiny neuron (MSN) activity (Witten et al., 2010). Hence, it is not surprising that the neurotransmitter acetylcholine has repeatedly been implicated in learning, decision making and reward functions (Grasing, 2016; Tian et al., 2016).

Yet, acetylcholine seems to have modulatory, rather than directly reinforcing, properties.

For instance, neither optogenetic activation nor inhibition of cholinergic interneurons in the NAc is capable of inducing place preference or avoidance (Lee et al., 2016), whereas optogenetic inhibition of NAc cholinergic interneurons attenuates the acquisition of cocaine conditioned place preference (CPP; Witten et al., 2010).

Acetylcholine has been suggested to play a role for reinforcement-learning specifically associated with drugs of abuse. It has been demonstrated that rats display significant increases in accumbal acetylcholine during the learning of a drug-of-abuse operant runway model. The acetylcholine increase was specific to drugs of abuse (i.e., morphine, remifentanil and cocaine) and did not occur during learning with palatable food (Crespo et al., 2006, 2008). This is in line with the finding that activating cholinergic interneurons in the NAc during extinction promotes the loss of place preference towards cocaine but leaves extinction of food CPP unaffected (Lee et al., 2016).

Acetylcholine binds to two types of receptor-families categorized according to their selectivity towards specific pharmacological ligands: the nicotinic and the muscarinic acetylcholine receptors. Muscarinic receptors are G-protein-coupled receptors and exist in five subtypes. The M1, M3 and M5 receptors are excitatory receptors, whereas M2 and M4 receptors (M4Rs) inhibit neuronal firing by decreasing the intracellular concentration of cAMP (Langmead et al., 2008) and activating G-protein coupled inward rectifying potassium channels (Wess et al., 2007).

Muscarinic M4Rs are particularly relevant for controlling synaptic acetylcholine tone in the striatum and VTA, as they provide feedback-inhibition of transmitter release from the presynaptic cholinergic neurons (Zhang et al., 2002; Tzavara et al., 2004). The M4Rs are highly expressed throughout the forebrain, with the highest expression-levels occurring in the striatum as shown with *in situ* hybridization (Allen Brain Atlas^1^). Here they have been found to co-localize principally with the dopamine 1 receptor (D1R)-expressing MSNs (Jeon et al., 2010). Hence, it is not surprising that M4Rs were previously shown to play a role in the response to drugs of abuse. Global knock-out of M4Rs leads to enhanced cocaine and alcohol self-administration in mice (Schmidt et al., 2011; de la Cour et al., 2015). Furthermore, the conditional knock-out of M4Rs from D1R-expressing neurons caused a phenotype related to that of the global receptor deletion model, as these mice displayed increased locomotor responses to psychostimulants, as well as an elevated dopamine efflux in the striatum upon amphetamine injections (Jeon et al., 2010). However, the function of the specific M4R subpopulations, as auto-receptors on cholinergic neurons vs. post-synaptic receptors on D1R-expressing neurons, have not been explored in reward-learning.

Our study aimed to elucidate the relation between muscarinic M4Rs on dopamine D1-receptor (D1R) expressing neurons and those on cholinergic neurons, in acquisition of both drugs of abuse and natural reward reinforcement behaviors, as well as in impulsivity. For this purpose, we tested conditional knock-out mice in CPP and in an operant runway-paradigm for both palatable food and cocaine, as well as in drug primed reinstatement of cocaine place preference. We also investigated these mice in cocaine induced locomotor sensitization. We studied the effect of conditional M4R deletion on immediate early gene (IEG) expression in the forebrain after repeated cocaine exposure. Finally, we explored the role of the M4Rs on D1R neurons in the 5-choice-serial-reaction-time-test task (5CSRTT).

## Materials and Methods

### Animals

All animals in this study were male and their age was 8–20 weeks. Chrm4-floxed and D1RCre lines are described in the literature (Jeon et al., 2010) and ChATCre mice were purchased from The Jackson Laboratory. Mice were on a C57BL/6N background with minor (<15%) contributions from 129SvEv and C57BL/6J. Wildtype littermate animals carrying two floxed alleles of the M4R were used as controls. The control mice did not display significant differences in baseline (BL) behaviors in the place preference and operant runway tests and were therefore pooled to one control group in these experiments. Mice were single-housed 48 h prior to experiments, housed with environmental enrichment and kept in a pathogen-free facility on a regular 12-h light/dark cycle. All experiments were performed during the light phase. Food and water were provided *ad libitum* with the exception of animals in the 5CSRTT and the food-reward operant runway (as detailed in the respective “Materials and Methods” section). The use of animals for this study followed the EU directive 2010/63/EU for animal experiments and were approved by the Research Animal Care and Use Committee in Linköping, Sweden.

### Drugs

Cocaine HCl was obtained commercially from Sigma-Aldrich and the Hässleholm Hospital Pharmacy, Sweden and dissolved in saline. Mice received 15 mg/kg cocaine unless otherwise specified.

### Locomotion

Locomotion was monitored in a standardized locomotor chamber box (450 [W] × 450 [D] × 400 [H] mm) divided in 4 equal-sized compartments from Panlab, Harvard Apparatus. The locomotor activity of 4 mice was monitored simultaneously over 30 min using SMART VIDEO TRACKING software (Panlab, Harvard Apparatus). On day 0 mice received an intraperitoneal (i.p.) saline injection immediately before being placed into the tracking box. From day 1 to day 6 mice received 15 mg/kg cocaine i.p. immediately before video-tracking. Subsequently, the mice were left in their home-cages for 14 days. On day 20, the mice were placed back into the tracking box without any injection to monitor conditioned locomotor activity. On day 21, the mice received a final injection of 15 mg/kg cocaine and were video-tracked, in order to study drug-induced sensitization effects.

### Conditioned Place Preference

We used a place conditioning procedure to measure preference, applying a 3-chambered Panlab Spatial Place Preference Box (Harvard Apparatus), according to previously published protocol (Klawonn et al., 2017). On day 1, during a 15-min pretest, the individual mouse was allowed to move freely between the chambers of the box. Time spent in each compartment was manually recorded by two independent experimenters blinded to genotype. To ensure explorative behavior during the pretest, each mouse had to cross the corridor, entering the opposing chamber a minimum of five times to be included in the experiment. Any animal that spent >66% of their time during pretests in either of the conditioning chambers were discarded from the study. Mice were assigned to vehicle- or cocaine-paired compartments in a manner to avoid reinforcing natural bias, i.e., cocaine injections were paired with the least preferred chamber identified during pretest. This method has been shown to produce reliable CPP responses to cocaine (Fritz et al., 2016). On day 2 in the morning, mice were injected with saline i.p. before confinement for 15 min in the vehicle chamber. Four hours later on the same day, the mice were trained to cocaine (15 mg/kg) i.p. in the opposite chamber. This alternating training procedure was continued for four consecutive days, until day 6, when the CPP was assessed by allowing the mice to freely explore all compartments of the box for 15 min. The individual preference score was calculated by subtracting the time the mouse spent in the cocaine-paired chamber during the pretest from that of the posttest. The conditioning boxes were thoroughly cleaned after each mouse using warm water and a surface disinfectant.

In order to study reinstatement behavior, mice which expressed place preference towards cocaine underwent 6 days of extinction training. During extinction, the training sessions remained the same, except mice were given saline i.p. in both conditioning and vehicle chambers. Twenty-four hours after the last extinction training-session; the mice underwent an extinction test for 15 min. Mice that lost >60% of their initial preference were allowed to proceed to the reinstatement-session 24 h later. To reinstate drug-seeking, the mice received 5 mg/kg cocaine i.p. immediately before a 15-min reinstatement test. The reinstatement score was calculated as time spent in the cocaine-associated chamber during the extinction test subtracted from that during the reinstatement test.

For natural reward place preference, we employed the same conditioning procedure as for cocaine. A small amount of Nutella (Ferrero©) on a gray tile was used as the unconditioned stimulus during training, compared to a clean gray tile in the non-conditioning chamber.

### Catheter Surgery

Catheterization was performed under anesthesia induced by a mixture of 1 mg/kg dexmedetomidine and 75 mg/kg ketamine i.p. using aseptic surgical techniques. Following induction, the mice received i.p. analgesia (0.1 mg/kg buprenorphine). A catheter (MIVSA mouse catheter, CamCaths) with a 9.0-cm length of silicone tubing (inner diameter, 0.2 mm; outer diameter, 0.4 mm) was inserted 1.2 cm into the right jugular vein, tunneled s.c. to the ventral aspect of the neck, and anchored to a 26-gauge stainless steel tubing in plastic secured s.c. with a propylene knitted mesh (diameter, 20 mm). Catheter patency was confirmed by backflush, and skin incisions were closed with Prolene 6-0 (Johnson & Johnson). Anesthesia was reversed with atipamezol (1 mg/kg) s.c., and the mice were left to recover in a euthermic environment. After surgery, mice were given i.p. analgesic (buprenorphine, 0.1 mg/kg) at least every 12th hour for 2 days.

### Operant Runway

For operant reinforcement, we used a custom-made mouse-runway built by AgnThos AB. The runway consisted of two chambers measuring 14 cm × 14 cm × 25 cm and equipped with retractable doors connected by a gray corridor (8 cm × 80 cm × 25 cm). The start-chamber was white, while the goal-chamber had black on white dotted wall-paper, black floor and a cue-light. For the palatable food runway, mice were habituated to Nutella^®^ in their home-cages for 2 days and food-restricted 4 h prior to the experiment. At the start of each trial (run), a small quantity Nutella^®^ was placed in the goal-chamber. Each mouse was given five consecutive runs separated by 1 h, which was spent in the home-cage. The time needed for an animal to obtain the reward (runtime) is considered inversely proportional to the strength of that specific stimulus (Wakonigg et al., 2003; Ettenberg, 2004). Mice that didn’t reach the goal-chamber within 90 s were gently guided there. Runtimes were manually recorded by two independent experimenters blinded to genotype. After entering the goal-chamber, the door was closed and the mouse was confined there for 2 min, while the cue-light was on. The runway was cleaned with a surface disinfectant between each run.

For cocaine conditioned runway, mice underwent intravenous catheterization minimum 48 h prior to the test. Catheters were flushed daily with 100 μl heparin to ensure proper flow. PE-tubing connected to a 300 μl syringe was attached to the catheter-outlet on the mouse’s back. Mice were acclimatized to the tubing for 10 min prior to the test. The cocaine-enforced runway followed the identical protocol as the food reward-enforced runway, with the exception that the inter-trial-interval (ITI) was 15 min. During which, mice spent 5 min in the goal-box followed by 10 min in a separate resting-chamber. Upon entrance into the goal-box, mice received 0.3 mg/kg cocaine over 6 s with cue-light on. Following the runway-test, 100 mg/kg pentobarbital was injected to control for catheter patency and euthanizing the animals.

### 5-Choice-Serial-Reaction-Time-Task

The 5CSRTT was used to assess impulsivity. Three weeks before training-start, the animals were food-restricted to achieve 85% ± 5% of free-feeding weight. During this time, mice were habituated to the reward (raspberry-juice) in their home-cages for 3 days. Behavioral training was performed using Bussey-Saksida Touchscreen Chambers (Campden Instruments, UK) with the 5CSRTT mask (illustration in Figure 4). Each conditioning-chamber is housed inside a sound-attenuating box. Chambers are trapezoidal with three black walls. The front wall of the chamber is a touchscreen monitor, covered with a black plastic mask with five response-windows, thereby limiting the response-area to the region where the visual stimulus (VS) is displayed. A reward magazine can be accessed by the mouse via a window in the wall opposite the touchscreen. Juice reward (7 μL, unless noted differently) was delivered to the magazine using a peristaltic pump.

**Figure 1:**
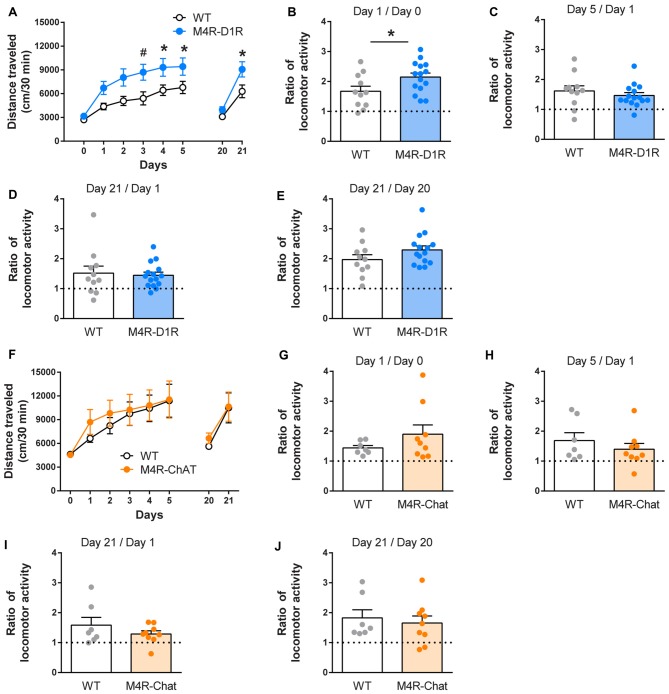
Cocaine-induced locomotor responses are elevated in muscarinic M4 receptor (M4R)-D1RCre mice. M4R-D1RCre mice increased their locomotor response to repeated 15 mg/kg cocaine injections (i.p.) to a greater degree than wildtype littermate controls **(A)** (*n* = 11 WT, *n* = 15 M4R-D1R). Acute cocaine-induced locomotion was increased in M4R-D1RCre mice compared to controls **(B)**. Locomotor sensitization (the ratio between day 1 and 5 or between day 1 and 21) remained unchanged **(C,D)**, this was also the case for cocaine-induced locomotion after the sensitization protocol **(E)**. In contrast, the locomotor response of M4R-ChATCre mice did not differ from WT mice at any time point **(F–J)** (*n* = 7 WT, *n* = 9 M4R-ChAT). *p < 0.05 repeated measure one-way analyses of variance (ANOVA) followed by Bonferroni’s *post hoc* test **(A)** or Student’s unpaired *t*-test **(B)**, ^#^*p* < 0.05 two-way ANOVA followed by Bonferroni’s *post hoc* test for genotype difference **(A)**.

**Figure 2:**
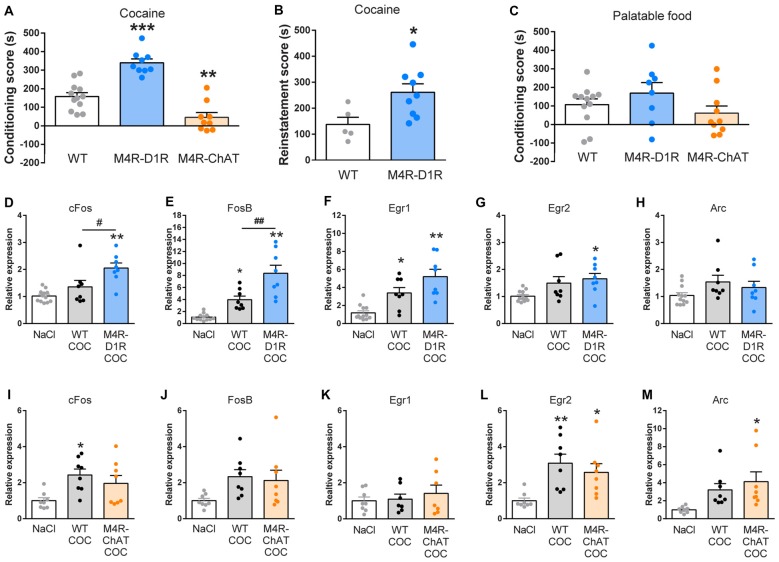
M4R-D1RCre and M4R-ChATCre mice exhibit opposite responses to Pavlovian conditioning, consistent with upregulated immediate early genes (IEGs) in forebrain of M4R-D1RCre mice after repeated cocaine exposure.** (A)** M4R-D1RCre mice displayed stronger cocaine-seeking compared to wildtype littermate control mice, whereas M4R-ChATCre mice failed to develop cocaine-induced conditioned place preference (CPP; *n* = 12 WT, *n* = 9 M4R-D1RCre, *n* = 8 M4R-ChATCre). **(B)** Deletion of M4Rs from dopamine D1 receptor (D1R)-expressing neurons significantly increased reinstatement in response to 5 mg/kg cocaine i.p. (*n* = 5 WT, *n* = 9 M4R-D1RCre). **(C)** Neither M4R-D1RCre nor M4R-ChATCre mice differed significantly from wildtype littermate control mice in a palatable food-enforced Pavlovian place conditioning paradigm (*n* = 12 WT, *n* = 8 M4R-D1RCre, *n* = 10 M4R-ChATCre).** (D–H)** Comparisons of IEG expression in the forebrain of saline controls (NaCl; *n* = 12), cocaine exposed controls (WT COC; *n* = 8) and cocaine exposed M4R-D1RCre (M4R-D1RCre COC; *n* = 8) mice. **(D)** *cFos* was significantly upregulated in M4R-D1RCre mice compared to saline control and cocaine control mice. **(E)** Both M4R-D1RCre and control mice displayed an elevation of *FosB* compared to saline control mice, which was significantly augmented in M4R-D1RCre mice. The IEGs *Egr1* and *Egr2* were significantly induced by cocaine in M4R-D1RCre mice, but only *Egr1* was elevated in control mice that received cocaine **(F,G)**. No changes were observed in the expression of *Arc* upon cocaine stimulation **(H)**. **(I–M)** Comparisons of IEG expression in the forebrain of NaCl (*n* = 8), WT COC (*n* = 8) and M4R-ChATCre COC (*n* = 8). M4R-ChATCre mice lacked a significant induction of *cFos*, *FosB* and *Egr1* post cocaine, whereas control cocaine mice displayed a significant upregulation of *cFos* in comparison to saline controls **(I–K)**. *Egr2* **(L)** was significantly elevated by cocaine independently of the genotype, while *Arc* **(M)** was only induced by cocaine in M4R-ChATCre mice. *p < 0.05, **p < 0.01, ***p < 0.001 Student’s paired *t*-test or one-way ANOVA followed by Bonferroni’s *post hoc* test (in the qPCR results this indicates differences relative to saline controls). ^#^*p* < 0.05, ^##^*p* < 0.01 (indicates qPCR differences between cocaine-groups) one-way ANOVA followed by Bonferroni’s *post hoc* test.

**Figure 3:**
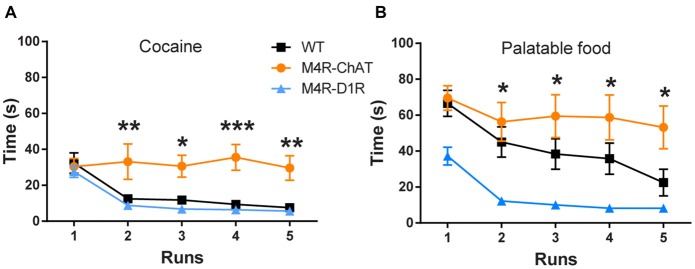
M4R-ChATCre mice are unable to learn operant responding to reward.** (A)** Both M4R-D1RCre and wildtype littermate control mice learned the operant runway paradigm for cocaine (0.3 mg/kg i.v.; *n* = 13 WT, *n* = 5 M4R-D1RCre, *n* = 8 M4R-ChATCre) and **(B)** palatable food (*n* = 10 WT, *n* = 6 M4R-D1RCre, *n* = 6 M4R-ChATCre). M4R-ChATCre mice were unable to acquire an operant response to neither palatable food, compared to M4R-D1RCre, nor cocaine, compared to both controls and M4R-D1RCre mice **(A,B)**. *p < 0.05, **p < 0.01, ***p < 0.001 two-way ANOVA followed by Bonferroni’s *post hoc* test.

**Figure 4:**
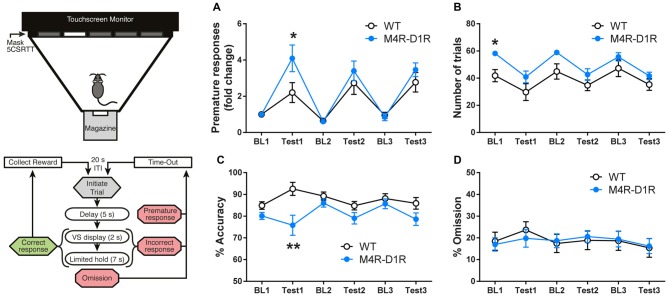
M4R-D1RCre mice exhibit impulsive behavior in the 5-choice-serial-reaction-time-task (5CSRTT). As illustrated in the flow-chart, each trial was initiated by a magazine nose-poke, followed by a 5-s Delay before visual stimulus (VS) display. Touching a window during the Delay was scored as *Premature Response (PR)* and resulted in Time-out (illumination of the chamber lamp for 5 s) followed by repeat of the same trial. Touches to the window displaying the VS were scored *Correct* (resulting in Reward), while touches to other windows were scored *Incorrect* (resulting in Time-out). If the mouse failed to respond during VS display, the trial entered a Limited Hold period, during which Correct or Incorrect touches were still applicable. Failure to respond during this time was scored as an *Omission* and resulted in Time-out. A 20-s inter-trial interval (ITI) followed each reward collection or Time-out. Summary of performance during the 5CSRTT showing mean values (*n* = 7 WT, *n* = 8 M4R-D1RCre) during three sequential baseline (BL) and impulsivity-testing (TEST) conditions. Parameters assessed were trials/session **(A)**, PRs (as fold change from BL1; **B**), % accuracy **(C)**, and % omissions **(D)**. **(A)** During BL conditions M4R-D1RCre mice learn the 5CSRTT paradigm better than wildtype littermate control mice, as reflected in a significantly increased number of rewards earned per session. M4R-D1RCre mice showed a significantly increased number of PRs during Test 1 **(B)**. In line with the increase in PRs, M4R-D1RCre mice were also less accurate during Test 1 **(C)**. Omissions did not differ between the two genotypes **(D)**. *p < 0.05, **p < 0.01 two-way ANOVA followed by Bonferroni’s *post hoc* test.

Each mouse was habituated to its touchscreen-chamber for 20 min (Day 1) and 30 min (Day 2), during which approximately 1 ml of juice was in the magazine at session-start and more juice was administered 30 s after nose-pokes to the magazine. Then training progressed through three pre-training categories and four 5CSRTT-training stages (parameters summarized in Table 1). *Initial Touch* trials consisted of two sessions of 30 min each, during which a 30 s display of the VS (illumination of a response window) was followed by juice delivery, accompanied by reward cues (a 3-kHz tone and magazine illumination). Juice-retrieval initiated an ITI of 5 s before display of the next VS. VS location alternated pseudo-randomly (balanced across the five windows and never appearing in the same location >3× in a row). If the mouse touched the VS while displayed, an immediate triple reward (21 μl juice) was delivered accompanied by reward-cues.

**Table 1:** Stages of 5-choice-serial-reaction-time-task (5CSRTT) training.

Training stage	ITI (s)	Delay (s)	VS duration (s)	Limited hold (s)	Session duration	Criterion
Initial touch	5	0	30	N/A	30 min
Must touch	5	0	until touched	N/A		2 Sessions
Must initiate	5	0	until touched	N/A		2 Sessions with >30 trials in 1 session
5CSRTT Stage 1	20	5	16	21	Maximum: 60 trials or 30 min whichever is first	>40 Trials+ >80% Accuracy+ <20% Omissions in 3 of 4 sessions
5CSRTT Stage 2	20	5	8	13		
5CSRTT Stage 3	20	5	4	9		≥50 Trials+ 80% Accuracy+ <20% Omissions in 3 of 4 sessions
5CSRTT Stage 4	20	5	2	7		
5CSRTT baseline	20	5	2	7	
5CSRTT test	20	10	2	7	Maximum: 60 trials or 60 min	N/A

*Summary of the progression in inter-trial interval (ITI), Delay, visual stimulus (VS) duration, limited hold, session duration and completion criterion across the three pre-training categories and the four stages of 5CSRTT training*.

*Must Touch* sessions proceeded as above, except the mouse was required to touch the VS before reward would be delivered. The VS display was not time-limited but terminated upon touch.

During *Must Initiate* sessions, mice also had to initiate each trial. Following the ITI, the magazine was illuminated (without juice-delivery) and a subsequent nose-poke initiated a click cue and the display of the VS. In order for the mice to proceed to the *5CSRTT Training trials* they had to initiate more than 30 trials in one of the two sessions.

#### 5CSRTT Training Trials

Trials were initiated by a magazine nose-poke, followed by a 5 s Delay before the VS was displayed. Touching any window during the Delay was considered a *Premature Response (PR)* and resulted in a Time-out and repeat of the trial. The VS was displayed for a set duration (Stimulus Duration), during which touches to the VS window were scored as *Correct* (resulting in Reward), while touches to any other window were scored as *Incorrect* (resulting in a Time-out). If the mouse failed to respond to any window during VS display, the trial entered a Limited Hold period during which Correct or Incorrect touches were still applicable. Failure to respond to any window during the Limited Hold was scored as an *Omission* and resulted in a Time-out followed by advancement to the next trial.

A 20 s ITI followed each reward collection or Time-out before the next trial could be initiated by the mouse. Each session was limited to a maximum of 60 trials or 30 min. 5CSRTT-training progressed through four stages during which the Stimulus Duration and Limited Hold were reduced as outlined in Table 1. To summarize, Trials resulting in Time-outs were *PRs, Incorrect Responses* (IR), or *Omissions* (OM). Only *Correct Response* (CR) trials resulted in Reward. Two measures were used to assess session performance: *% Accuracy* = CR/(CR + IR); and *% Omission* = OM/(OM + CR + IR).

#### 5CSRTT Baseline and Test Trials

After passing criterion on Stage 4 of 5CSRTT-training (described in Table 1), each mouse performed seven sessions with Stimulus Duration fixed at 2 s and a Delay of 5 s (max trials limited to 60 and max time of 30 min) to establish BL performance (BL1 in Figures 4A–D). Impulsivity was assessed across 3 Tests sessions, each separated by a minimum of three BL sessions (displayed as averages, BL2 and BL3 in Figures 4A–D). During Test-sessions all parameters of the 5CSRTT were the same as during BL (schematic illustration in Figure 4), except the max time was extended to 60 min and the Delay was increased to 10 s.

### Quantitative PCR

Brain tissue was used for qPCR analysis. Mice received one daily i.p. injection of 15 mg/kg cocaine or saline over four consecutive days. One hour after the last injection, mice were humanely euthanized by asphyxiation with CO2. A coronal section of 2 mm, posterior to the olfactory bulb, was removed using a brain matrix in ice cold miliQ-water, and the samples were immediately frozen in liquid nitrogen. In a supplemental experiment the striatum was needle-dissected from forebrain sections before freezing. Sections were homogenized in lysis buffer using a TissueLyser (QIAGEN) for 2 min at 20 Hz. RNA extraction was performed with the RNeasy Lipid Tissue Kit (Qiagen). cDNA was synthesized with High Capacity cDNA Reverse Transcription Kit (Applied Biosystems). Quantitative PCR was performed with Real-Time 7500 Fast apparatus (Applied Biosystems), using TaqMan Gene Expression Master Mix and TaqMan assay targeting *cFos* (Mm00487425_m1), *FosB* (Mm00500401_m1), *Arc* (Mm01204954_g1), *Egr1* (Mm00656724_m1) or *Egr2* (Mm00456650_m1). *GAPDH* (Mm99999915_g1) was used as endogenous control. Relative quantification was done by ΔΔCT method.

### Statistics

Results are illustrated as mean ± SEM. When comparing more than two groups with comparable variances, one- or two-way analysis of variance (ANOVA) was used, followed by *post hoc* analysis with Bonferroni’s multiple comparison tests to evaluate pairwise group differences. In cases of comparisons between two groups an unpaired Student’s *t*-test was applied. *P* < 0.05 was considered statistically significant. The program GraphPad Prism 6^®^ was used for statistical analysis.

## Results

### Locomotor Sensitization

To investigate how the auto-receptor population of M4Rs differ from the postsynaptic M4Rs expressed on D1R-neurons in drug-related behaviors, we started out by studying their role in cocaine induced locomotor sensitization. Mice lacking M4Rs on D1R expressing neurons did not differ from littermate controls during BL locomotion (Figure 1A). But when comparing the acute effect of cocaine between control and knockout animals, the M4R-D1RCre mice displayed an increased locomotor response (Figure 1B; Students’ unpaired *t*-test *p* < 0.05). Both WT mice and M4R-D1RCre mice developed locomotor sensitization after repeated injections of cocaine (Figures 1C,D paired *t*-test comparing day 1 and 5, WT; *p* < 0.01, M4R-D1RCre; *p* < 0.001 and day 1 and 21: WT; *p* < 0.01 and M4R-D1RCre; *p* < 0.001). There was no significant difference in the magnitude of the locomotor sensitization between the genotypes (Figures 1C,D Students’ unpaired *t*-test). Finally, cocaine increased the locomotor activity in both WT and M4R-D1RCre mice after the sensitization protocol (Figure 1E; Students’ paired *t*-test comparing day 20 and 21; *p* < 0.05 in both genotypes). M4R-ChATCre mice did not differ from WT mice in their basal locomotion (Figure 1F). Cocaine induced an acute locomotor response in both M4R-ChATCre and WT mice (Figures 1F,G; *p* < 0.001 in WT and *p* < 0.05 in M4R-ChATCre mice using Students’ paired *t*-test) that did not differ between genotypes (Students’ unpaired *t*-test). The WT mice displayed a statistically significant sensitization (Students’ paired *t*-test comparing day 1 and 5; *p* < 0.05 and day 1 and 21; *p* < 0.05) whereas the M4R-ChatCre mice only showed a strong tendency towards sensitization (day 5; *p* = 0.057 and day 21; *p* = 0.050, compared to day 1 using Students’ paired *t*-test). A direct comparison between the sensitization responses in WT and M4R-ChATCre mice showed no statistically significant difference (Figures 1H,I; comparing ratios of day 5 and 1; *p* = 0.39 and day 21 and 1; *p* = 0.32 using Students’ unpaired *t*-test). After the sensitization protocol, cocaine increased locomotor activity in both genotypes (Figure 1J; Students’ paired *t*-test comparing day 20 and 21; *p* < 0.05 in both genotypes).

### Pavlovian Conditioning to Natural Reward and Cocaine

In order to explore the role of M4Rs in natural and drug-of-abuse related reward learning, we employed a place preference paradigm towards palatable food (Nutella^®^) or cocaine. M4R-D1RCre mice showed a significantly increased CPP towards cocaine (Figure 2A; one-way ANOVA *F*_(2,27)_ = 38.33 *p* < 0.001) and a 5 mg/kg cocaine priming dose resulted in a significantly increased re-introduction of cocaine-seeking behavior after extinction in these mice compared to controls (Figure 2B; Students’ unpaired *t*-test *p* < 0.05). M4R-ChATCre mice, on the other hand, did not display intact cocaine seeking behavior towards the cocaine-paired compartment, resulting in a considerably lowered CPP-score than the controls (Figure 2A; one-way ANOVA *F*_(2,27)_ = 38.33 *p* < 0.001). Although, preference scores to food remained unchanged in both M4R-D1RCre and M4R-ChATCre mice, they showed similar tendencies as in the cocaine place preference paradigm (Figure 2C).

### Expression of Immediate Early Genes

To evaluate the roles of the two specific M4R populations for the expression of IEGs involved in neural activity, plasticity and motivation (Minatohara et al., 2016), we investigated expression of *cFos*, *FosB*, *Egr1*, *Egr2* and *Arc* after four 15 mg/kg cocaine injections over four consecutive days. The induction of IEGs was relatively moderate, as might be expected given that these animals received multiple cocaine injections and samples included the complete forebrain. Cocaine increased mRNA levels of several IEGs in control mice (Figure 2E; One-way ANOVA; *F*_(2,25)_ = 25.73 *p* < 0.001, Figure 2F; One-way ANOVA; *F*_(2,25)_ = 15.54 *p* < 0.001, Figure 2I; One-way ANOVA; *F*_(2,21)_ = 4.93 *p* = 0.01, Figure 2L; One-way ANOVA; *F*_(2,21)_ = 6.99 *p* < 0.01). Interestingly, after cocaine exposure M4R-D1RCre mice showed a significant increase in *cFos* (Figure 2D; One-way ANOVA; *F*_(2,25)_ = 11.10 *p* < 0.001) and *FosB* (Figure 2E; One-way ANOVA; *F*_(2,25)_ = 25.73 *p* < 0.001) compared to control mice. This observation is in line with the exacerbated cocaine-seeking phenotype displayed by the M4R-D1RCre mice. *Egr1* and *Egr2* were upregulated in M4R-D1RCre mice compared to WT NaCl mice, but no differences were observed between cocaine groups (Figures 2F,G), while Arc expression remained unchanged (Figure 2H). When investigating the role of the M4 auto-receptors in cocaine induction of IEGs, no significant differences were observed between the M4R-ChATCre and control mice (Figures 2I–M). Interpretation of the IEG experiments should be done with some caution since differences between different regions and cell-types cannot be distinguished with this approach. To investigate if repeated cocaine injections and involvement of other circuitry influenced the observed changes in expression of IEGs in M4R-D1RCre and M4R-ChATCre mice, we investigated c-fos mRNA levels in the striatum after an acute injection of cocaine. This experiment demonstrated the identical tendencies in c-fos expression levels according to genotype as was observed in the forebrain after four injections of cocaine. M4R-D1RCre mice displayed a significant induction of c-fos after cocaine compared to controls, while the M4R-ChATCre mice showed no significant differences (Supplementary Figure S1).

### Operant Responding to Natural Reward and Cocaine

We next assessed if the two M4R populations would also have disparate effects on operant reinforcement behaviors. To do so, we used an operant runway model with either palatable food or intravenously (i.v.) administered cocaine as unconditioned stimuli. Consistent with our findings in the CPP paradigm, M4R-ChATCre mice were unable to learn operant responses to either i.v. cocaine or palatable food, as they maintained the same runtime across all runs. In contrast, both control and M4R-D1RCre mice significantly decreased their runtime over the five consecutive runs to both cocaine and palatable food (Figure 3A; Two-way-ANOVA; *F*_(4,92)_ = 8.559 *p* < 0.001 (time) and *F*_(2,23)_ = 9.529 *p* = 0.001 (genotype), Figure 3B; Two-way-ANOVA; *F*_(4,100)_ = 10.98 *p* < 0.001 (time) and *F*_(2,25)_ = 6.675 *p* = 0.001 (genotype)).

### Impulsivity in the 5-Choice-Serial-Reaction-Time-Task

After observing the augmented effect of M4R-D1RCre knockout on reward-reinforced behaviors, we went on to examine if this specific population of M4Rs plays a role in impulsive behavior. M4R-D1RCre mice and their controls underwent a 5CSRTT paradigm. The M4R-D1RCre mice learned the challenging operant task better than control mice, as reflected by the significantly higher number of rewards obtained under BL conditions (Figure 4A; Two-way-ANOVA; *F*_(1,78)_ = 20.08 *p* < 0.001 (genotype)). However, during the first test session M4R-D1RCre mice showed a significant increase in PRs compared to control mice, which is indicative of a deficit in waiting impulse control (Figure 4B; Two-way-ANOVA; *F*_(1,78)_ = 4.623 *p* < 0.05 (genotype)). Interestingly, the M4R-D1RCre mice adapted to the test conditions, as PRs did not differ from those of controls during Test 2 and Test 3. In accordance with the exacerbated waiting-impulsivity, the percent accuracy during Test 1 was significantly decreased in the M4R-D1RCre mice relative to controls (Figure 4C; Two-way-ANOVA; *F*_(1,78)_ = 19.57 *p* < 0.001 (genotype)). Omissions (i.e., initiating a trial but failing to respond) did not differ between the two genotypes throughout the experiment (Figure 4D). Due to the advanced operant level of the 5CSRTT and the inability of the M4R-ChATCre mice to acquire a basic operant response, we chose not to test these mice in this paradigm.

## Discussion

In this study, we used a transgenic approach to distinguish the roles of specific neuronal populations of M4Rs in palatable food and cocaine reward behaviors, and in impulsivity. We focused two cellular subpopulations of M4Rs: M4Rs specifically controlling cholinergic neurons by negative feedback and postsynaptic M4Rs responsible for modulating the activity of D1R-expressing neurons. The conditional deletion of M4Rs from D1R-neurons induced an impulsive phenotype during the 5CSRTT and caused an increase in cocaine-seeking behavior and cocaine-induced reinstatement in a CPP paradigm, together with operant responding to food reward. However, the M4R-D1RCre mice did not differ from controls in their operant response to cocaine. A possible explanation for this observation could be the practical limitations of the runway paradigm. During cocaine conditioning both knockout and control animals decreased their runtimes to the minimum allowed by the length of the runway. To explore phenotype-specific decreases in runtime, it would be relevant to adjust either the length of the runway or extend the duration of the paradigm to several days in order to diminish the locomotor effects of the repeated cocaine injections. The M4R-D1RCre related behavioral phenotypes were accompanied by significant increases in IEG expression in the forebrain.

In contrast, a deletion of M4Rs from cholinergic neurons rendered mice unable to acquire any learning task, with the possible exception of food-reinforced Pavlovian conditioning.

It is clear from our study that the M4Rs on dopaminoceptive D1R-neurons play an important role in reducing the reinforcing effects of drugs-of-abuse, and possibly natural rewards as observed during BL reward learning in the 5CSRTT.

The age of the animals used in the present study was 8–20 weeks, as breeding in some cases was slow and required us to wait in order to ensure complete experimental groups. Age-differences could influence behavioral responses to reward, but we find it unlikely to have affected the results of our study since the majority of animals were in the middle of the age-interval and the age was balanced between groups in the experiments. Furthermore, no obvious differences were observed between mice of different ages in the assays used in the present study. This is in line with previous reports demonstrating that US-CS learning is intact in C57BL6 mice of a similar age as in our experiments (Kaczorowski and Disterhoft, 2009; Harb et al., 2014).

We found, in agreement with a previous study on M4Rs (Jeon et al., 2010), that absence of this receptor population led to increased cocaine induced locomotor responses. This is interesting, as locomotor sensitization is believed to be a physiological consequence of synaptic changes occurring within the dopaminergic system in response to drugs-of-abuse (Steketee and Kalivas, 2011). This finding is in accordance with previous observations that the M4R-D1RCre mice exhibit elevated dopamine-release in the striatum (Jeon et al., 2010). Hence, it is likely that the exacerbated reward-phenotype following M4R deletion from D1R-neurons is a consequence of increased dopaminergic transmission. It was previously shown that D1R-MSNs of the NAc, projecting to GABAergic interneurons in the VTA, are involved in cocaine induced disinhibition of VTA dopamine neurons (Bocklisch et al., 2013). Hence relief of inhibition of the NAc D1R-MSNs, by removal of the inhibitory M4Rs, could be a mechanism involved in the augmented dopamine and reinforcement response of these mice. The finding that markers of neuronal activity, *cFos* and *FosB*, were significantly elevated in the striatum and forebrain of these mice (compared to controls) following cocaine exposure, would support this concept.

Such changes in mesolimbic neurotransmission could also explain the impulsive phenotype observed in the 5CSRTT paradigm. The involvement of dopamine in impulsivity has been established in healthy human beings (Buckholtz et al., 2010), and psychopathologies characterized by increased impulsivity, such as drug addiction, are accompanied by altered dopaminergic transmission (Volkow et al., 2001; Ersche et al., 2010).

The M4Rs on D1R-expressing neurons could be interesting as pharmacological targets, as they exert control over a broad array of behaviors related to addiction pathology; including locomotor sensitization, impulsivity, drug seeking and reinstatement of drug-seeking behavior after extinction. In line with this, a recent study on genetic polymorphisms within the M4R gene revealed a link to both cocaine and heroin addiction in human subjects (Levran et al., 2016).

It is clear from our study that the M4 auto-receptors responsible for negative regulation of acetylcholine release are pivotal for reward learning in both Pavlovian and operant tasks. The previously discussed limitations of the runway test do not pose a problem in the case of the M4R-ChATCre mice as these exhibit unchanged run-times, i.e., lack of learning. The run-times cannot be explained by general differences in cocaine-induced locomotion, since the mutant mice display normal responses to cocaine. Interestingly, the inability of the M4R-ChATCre mice to respond to positive reinforcement may be independent of the motivational neurocircuitry. The absence of effects on cocaine-induced locomotor sensitization and on IEG expression in the forebrain and striatum, indicates that the M4R-ChATCre phenotype is unlikely to be a direct consequence of altered dopaminergic signaling. Hence, the specific neurocircuitry involved in the lack of reinforcement learning during absence of M4-autoreceptors remains to be explored. Our results are in accordance with the idea that acetylcholine influences reward learning in a bell-shaped manner, where too little or too much transmitter in the synapse is unfavorable (Grasing, 2016). It is known that acetylcholinesterase inhibitors (which directly elevate acetylcholine levels) only exert their effect at lower doses, whereas higher concentrations impair learning (Braida et al., 1996, 1997; Baldi and Bucherelli, 2005). In accordance with these findings, several studies of cholinergic neuron functionality during reward learning have been contradictory, with results ranging from U-shaped curves arising from cholinergic receptor agonists, to opposing results from lesion and inhibition studies (Grasing, 2016).

Our data suggest that during learning of positive reinforcement, acetylcholine signaling needs to be precisely tuned. Loss of postsynaptic inhibition of D1R-neurons seems to contribute to an impulsive reward seeking phenotype, whereas a loss of presynaptic feedback inhibition causes learning deficits. Our findings can explain some dichotomies observed with M4R agonists and antagonists, as the two M4R populations examined in our study mediate opposite functions. Our results suggest a potential role for the M4Rs in treating pathologies related to reward learning, such as drug addiction. The true challenge for the future will be to develop and fine-tune pharmacological treatments that can act primarily on specific receptor populations.

## Author Contributions

AMK and MF conceived the study and designed it with input from DE. AMK performed the locomotor measurements. AMK with the help of MF conducted the CPP and operant runway experiments. MF and SHL designed and analyzed data from the 5CSRTT experiment. MF conducted the 5CSRTT with assistance from AMK, AKS and SHL. DBW performed catheterization surgeries together with MJ. Tissue extraction and dissection for qPCR was performed by AMK with the help of DE. MF and MJ ran the qPCR experiments. Genotyping was done by MJ. JW provided the Chrm4-floxed and D1RCre mouse lines for the study. The manuscript was written by AMK and MF. All authors read, consented to and commented on the manuscript.

## Conflict of Interest Statement

The authors declare that the research was conducted in the absence of any commercial or financial relationships that could be construed as a potential conflict of interest.

## Footnotes

^1^http://mouse.brain-map.org/experiment/show/261
