# Supplementary figures and images for: Muscarinic M4 Receptors on Cholinergic and Dopamine D1 Receptor-Expressing Neurons Have Opposing Functionality for Positive Reinforcement and Influence Impulsivity
Source: Front Mol Neurosci. 2018 Apr 24;11:139. doi: 10.3389/fnmol.2018.00139 (PMC5928231; doi:10.3389/fnmol.2018.00139)

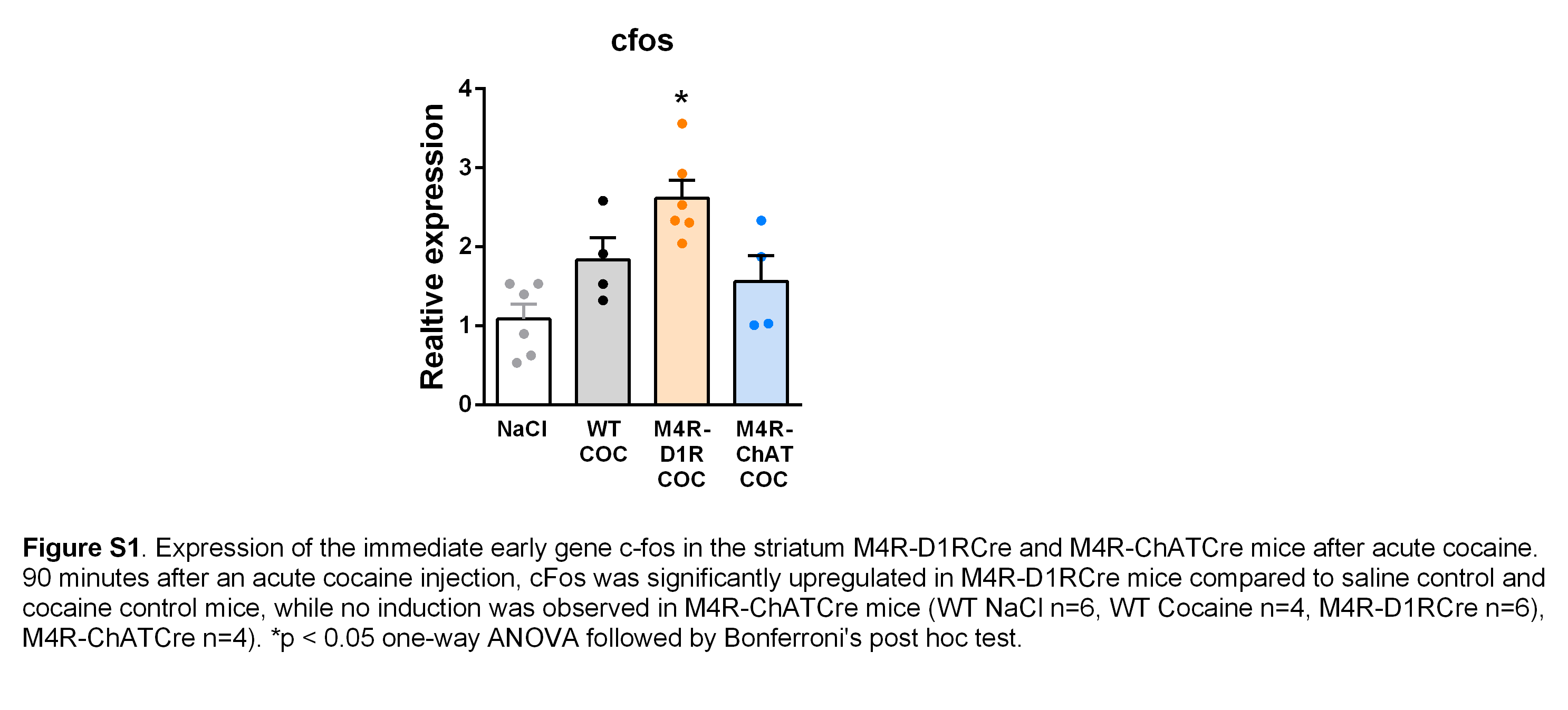

Supplement: Supplementary file 1 [file Image_1.TIFF]
